# Comparison of Fresh Frozen Tissue With Formalin-Fixed Paraffin-Embedded Tissue for Mutation Analysis Using a Multi-Gene Panel in Patients With Colorectal Cancer

**DOI:** 10.3389/fonc.2020.00310

**Published:** 2020-03-13

**Authors:** Xian Hua Gao, Juan Li, Hai Feng Gong, Guan Yu Yu, Peng Liu, Li Qiang Hao, Lian Jie Liu, Chen Guang Bai, Wei Zhang

**Affiliations:** ^1^Department of Colorectal Surgery, Changhai Hospital, Shanghai, China; ^2^Department of Nephrology, Changhai Hospital, Shanghai, China; ^3^Department of Pathology, Changhai Hospital, Shanghai, China

**Keywords:** colorectal cancer, multi-gene panel, gene mutation, fresh frozen tissue, formalin-fixed paraffin-embedded tissue

## Abstract

**Background:** Next generation sequencing (NGS)-based multi-gene panel tests have been performed to predict the treatment response and prognosis in patients with colorectal cancer (CRC). Whether the multi-gene mutation results of formalin-fixed paraffin-embedded (FFPE) tissues are identical to those of fresh frozen tissues remains unknown.

**Methods:** A 22-gene panel with 103 hotspots was used to detect mutations in paired fresh frozen tissue and FFPE tissue from 118 patients with CRC.

**Results:** In our study, 117 patients (99.2%) had one or more variants, with 226 variants in FFPE tissue and 221 in fresh frozen tissue. Of the 129 variants identified in this study, 96 variants were present in both FFPE and fresh frozen tissues; 27 variants were found in FFPE tissues only; 6 variants were found only in fresh frozen tissues. The mutation results demonstrated >94.0% concordance in all variants, with Kappa coefficient >0.500 in 64.3% (83/129) of variants. At the gene level, concordance ranged from 73.8 to 100.0%, with Kappa coefficient >0.500 in 81.3% (13/16) of genes.

**Conclusions:** The results of mutation analysis performed with a multi-gene panel and FFPE and fresh frozen tissue were highly concordant in patients with CRC, at both the variant and gene levels. There were, however, some important differences in mutation results between the two tissue types. Therefore, fresh frozen tissue should not routinely be replaced with FFPE tissue for mutation analysis with a multi-gene panel. Rather, FFPE tissue is a reasonable alternative for fresh frozen tissue when the latter is unavailable.

## Introduction

The survival of patients with colorectal cancer (CRC) has improved greatly in recent decades, because of advancements in surgical technique, chemoradiotherapy, and targeted therapy (1, 2). However, CRC remains the third most common cancer and the fourth cause of cancer-related death worldwide (3). Molecular biomarkers have been reported to play a vital role in the early diagnosis of CRC, and individualized treatment for metastatic CRC (4, 5). In the past decade, somatic gene mutations of *KRAS* (KRAS proto-oncogene, GTPase), *NRAS* (NRAS proto-oncogene, GTPase), and *BRAF* (B-Raf proto-oncogene, serine/threonine kinase) have been used to predict the outcomes of *EGFR* (epidermal growth factor receptor)—targeted therapy for metastatic CRC (4). Research studies of targeted therapy and individualized medicine have identified additional genes associated with the development and treatment of CRC (6). The classification of CRC based on multiple gene detection may help to explain inter-individual differences in treatment response and long-term outcomes. For example, the *UGT1A* (UDP glucuronosyltransferase family 1 member A complex locus) polymorphism has been reported to predict drug toxicity (delayed severe diarrhea) in patients with CRC who receive irinotecan-based chemotherapy (7, 8). Somatic mutations of *MLH1* (mutL homolog 1), *MSH2* (mutS homolog 2), *MSH6* (mutS homolog 6), and *PMS2* (PMS1 homolog 2, mismatch repair system component) in CRC tissue could be used to screen for Lynch syndrome (9, 10). Moreover, the detection of multiple gene mutations could offer more options for new targeted treatment efforts in drug-resistant patients (11). Hence, the need for combined detection of multiple gene mutations has acquired increasing urgency.

Next generation sequencing (NGS) is an efficient and rapid tool for the detection of single-nucleotide mutations and small-fragment insertion/deletions. This approach has become the standard technique for multiple gene detection (12). Compared with the single-gene mutation detection, multiple gene detection with NGS is a timely and cost-effective technique that requires a small amount of DNA (13, 14). In our previous study (15), we established a 22-gene panel, which included 103 amplicons targeting the variants found to be most common in CRC. Those 22 genes included *KRAS, BRAF, TP53* (tumor protein p53), *EGFR, CTNNB1* (catenin beta 1), *DDR2* (discoidin domain receptor tyrosine kinase 2), *ERBB2* (erb-b2 receptor tyrosine kinase 2), *ERBB4* (erb-b2 receptor tyrosine kinase 4), *FBXW7* (F-box and WD repeat domain containing 7), *FGFR1* (fibroblast growth factor receptor 1), *FGFR2* (fibroblast growth factor receptor 2), *FGFR3* (fibroblast growth factor receptor 3), *AKT1* (AKT serine/threonine kinase 1), *ALK* (ALK receptor tyrosine kinase), *MAP2K1* (mitogen-activated protein kinase kinase 1), *MET* (MET proto-oncogene, receptor tyrosine kinase), *NOTCH1* (notch receptor 1), *NRAS, PIK3CA* (phosphatidylinositol-4,5- bisphosphate 3-kinase catalytic subunit alpha), *PTEN* (phosphatase and tensin homolog), *SMAD4* (SMAD family member 4), *and STK11* (serine/threonine kinase 11). Use of this panel requires only 10 ng of DNA and a single-tube multiplex polymerase chain reaction (PCR).

Fresh frozen tissue is the preferred sample to detect gene mutation due to its superiority in preserving DNA. However, fresh frozen tissue is often not available in clinical practice, as the associated protocol requires that resected tissue be snap-frozen in liquid nitrogen 30–60 min after surgical resection. Moreover, the cost of preserving fresh frozen tissue is relatively high, as it requires the maintenance of a constant ultralow temperature. Compared with fresh frozen tissue, formalin-fixed paraffin-embedded (FFPE) tissue has several advantages: (1) preservation of the cellular and architectural morphology; (2) the possibility of storage at room temperature for several years; (3) easy availability, as FFPE blocks are routinely prepared in the pathology departments of most centers. Therefore, FFPE tissue has become the sample type used most commonly for molecular testing (11). However, the fixation and archiving process in FFPE often leads to the cross-linking, degradation, and fragmentation of DNA molecules. These alterations inevitably affect the quality and quantity of DNA extracted from FFPE tissue, which in turn would adversely impact the accuracy with which gene mutations are detected (16–19). Nevertheless, the detection of *EGFR* and *KRAS* mutations in DNA extracted from FFPE tissue has proven to be highly accurate (20). The detection of gene mutations using NGS may also be performed with DNA from FFPE samples (13, 14, 16, 21).

In our previous study, we showed the utility of this 22-gene panel when used with NGS to detect gene mutations in FFPE tissue from 207 patients with CRC (15). However, whether the gene mutation results of this multi-gene panel are concordant between FFPE and fresh frozen tissue remains unknown. In this study, gene mutations were detected in paired FFPE and fresh frozen primary tumor tissue from patients with CRC using this 22-gene panel. The results obtained were compared between tissue types at the variant and gene levels.

## Materials and Methods

### Inclusion Criteria

Patients who satisfied all of the following criteria were included: (1) pathologically proven primary CRC adenocarcinoma; (2) history of radical surgery for a primary tumor at Changhai Hospital (Shanghai, China) during the period from March 2015 to November 2016; (3) availability of FFPE and fresh frozen primary tumor tissue samples.

### Exclusion Criteria

Patients were excluded if they met any one of the following criteria: (1) history of preoperative radiotherapy; (2) insufficient FFPE or fresh frozen tumor tissue for the extraction of DNA; (3) history of local tumor excision; (4) recurrent CRC; (5) personal history of other tumors; (6) hereditary CRC.

### Patients

All patients with CRC satisfying the above-mentioned criteria were enrolled in the study. All the relevant clinicopathological information was prospectively maintained in an electronic CRC database. The study was approved by the ethical committee at Changhai Hospital. All included patients provided their written informed consent. All patients were followed up after surgery every 3 months for the first 2 years, every 6 months for the next 3 years, and every year from that point onward.

### Processing of Tumor Tissue

All tumor tissue samples were obtained from surgically resected primary CRC tumor specimens. The resected tissue was divided into two parts. One part was frozen in liquid nitrogen <30 min after surgery and stored at −80°C until the time of DNA extraction; the other part was fixed in 4% formalin for 24–72 h and embedded in paraffin, and then stored at room temperature. The blocks containing FFPE tissues were divided to obtain ten consecutive sections with thickness of 10 μm. Hematoxylin and eosin staining was performed on the first section. Two pathologists examined each stained section individually and estimated the percentage of neoplastic cells. Sections with ≥ 40% neoplastic cells were considered as eligible. The remaining nine sections were pooled into a 1.5-mL tube.

### DNA Isolation

The QIAamp DNA Mini Kit(Qiagen) and GeneRead DNA FFPE kit (Qiagen) were used to extract DNA from fresh frozen and FFPE tissues, respectively, according to the manufacturer's protocols. The detailed methods used for DNA extraction from FFPE tissue samples were reported previously by our group (15). Briefly, FFPE tissue samples were dewaxed with deparaffinization solution, then incubated with lysis buffer for 1 h. After incubation at 90°C to remove cross-links, Uracil-N-Glycosilase was added for the specific removal of deaminated cytosine residues from the DNA. For fresh frozen tissues, 25 mg of tumor tissues were used for DNA extraction. After being homogenized with the Bioprep-24 homogenizer, the tissues were incubated in lysis buffer at 56°C until the tissues were completely lysed. RNase A was used to digest the RNA molecules from the genomic DNA. For both FFPE and fresh frozen tissue, DNA was eluted and quantified with a Qubit 3.0 fluorometer and dsDNA HS assay kit (Life Technologies).

### DNA Amplification and Sequencing

For sequencing, 20 ng DNA extracted from each FFPE or fresh frozen sample was used for library construction. In brief, gene-specific PCR was used to amplify 103 regions in the first round, followed by purification via size selection. The details of the first round of PCR were showed in Table 1. Subsequently, the second round of PCR (“indexing PCR”) was conducted (Table 2). The 22-gene panel was purchased from Pillar Biosciences, USA (ONCO/Reveal Lung & Colon Cancer Panel). The details of the 22-gene panel were same as described previously (14, 15). This process involves the addition of Illumina index adaptors to purify the products for sample tracking and sequencing. Lastly, the libraries were eluted in 22 μL nuclease-free water. The final libraries were quantified using a Qubit 3.0 fluorometer and dsDNA HS assay kit (Life Technologies), as per the manufacturer's protocol. The MiSeq was used for sequencing libraries according to the manufacturer's protocol. Each gene library was normalized to 4 nM and combined at equal volume (4 μL). The mixed library was then denatured with 0.2 N NaOH and diluted up to the concentration of 15 pM for sequencing with MiSeq Reagent Kit v2 at 300 cycles (or 20 pM for v3). Sample quality of FFPE tissues was determined by the amount of amplifiable DNA using qPCR and sequencing libraries were evaluated using Bioanalyzer.

**Table 1:** The procedure details of the first round of PCR (“gene-specific PCR”).

**Temperature**	**Time**	**Cycles**
95°C	15 min	1
95°C	1 min	5
60°C	6 min	
95°C	30 s	18
72°C	3 min	
8°C	Hold	1

**Table 2:** The procedure details of the second round of PCR (“indexing PCR”).

**Temperature**	**Time**	**Cycles**
95°C	2 min	1
95°C	30 s	5
66°C	30 s	
72°C	60 s	
72°C	5 min	1
8°C	Hold	1

### Variant Analysis

Subsequently, they were annotated with the 1000 genomes (https://www.internationalgenome.org/home), which is one of the most frequently used databases in genetic research. Variants were selected for known single nucleotide polymorphisms (SNPs) and synonymous mutations. All non-coding, silent, synonymous, unknown and common germline variants were filtered out, as well as all variants present in 1,000 G data (22). Moreover, all variants at a locus with coverage of <200, or variants with a variant frequency <0.05 were excluded. The remaining mutations were assessed using the Catalog of Somatic Mutations in Cancer (COSMIC) database (14, 23). The SIFT (http://sift.jcvi.org/), PolyPhen-2 (http://genetics.bwh.harvard.edu/pph2/), and ClinVar (https://www.ncbi.nlm.nih.gov/clinvar/) online databases were used to analyze the clinical significance of these variants.

### Statistical Analysis

All data were analyzed using the Statistical Package for Social Sciences (SPSS 22.0, Chicago, IL). Categorical parameters were recorded as frequency and percentage; continuous parameters were described as mean and standard deviation, or median and interquartile range (IQR), as appropriate. Concordance rate and Kappa coefficient were used to compare FFPE and fresh frozen tissue in terms of mutation results, at both the variant and gene levels. *P* < 0.05 (two-sided) was considered as statistically significant.

## Results

### Clinicopathological Features of Patients Included in the Study

Of the 118 patients with CRC included in this study, 72 (61%) were men. The median age (IQR) was 62 (53–69) years. Most of the patients had TNM stage II (*n* = 36) or III (*n* = 47) disease. Eleven (9.3%) patients received preoperative chemotherapy (Table 3). The storage period of FFPE tissues ranged from 3 to 24 months, with a median period of 10 months.

**Table 3:** Clinicopathological characteristics of patients included in the study.

**Parameters**		***N*(%)**
Gender	Male	72 (61.0%)
	Female	46 (39.0%)
Gross type	Protruding	24 (20.3%)
	Ulcerative	91 (77.1%)
	Infiltrative	3 (2.5%)
Tumor position	Right sided colon cancer	26 (22.0%)
	Left sided colon cancer	40 (33.9%)
	Rectal cancer	52 (44.1%)
Differentiation	Well	0 (0.0%)
	Moderate	95 (80.5%)
	Poor	23 (19.5%)
T	1	2 (1.7%)
	2	17 (14.4%)
	3	81 (68.6%)
	4	18 (15.3%)
N	0	57 (48.3%)
	1	36 (30.5%)
	2	25 (21.2%)
M	0	95 (80.5%)
	1	23 (19.5%)
TNM	I	12 (10.2%)
	II	36 (30.5%)
	III	47 (39.8%)
	IV	23 (19.5%)
Tumor deposit	No	97 (82.2%)
	Yes	21 (17.8%)
Perineural invasion	No	106 (89.8%)
	Yes	12 (10.2%)
Vascular invasion	No	101 (85.6%)
	Yes	17 (14.4%)
Preoperative chemotherapy	No	107 (90.7%)
	Yes	11 (9.3%)
Age (years, IQR)		62 (53–69)
Diameter (cm, IQR)		4.1 (3.0–6.0)
CEA (ng/mL, IQR)		4.7 (2.0–15.4)
CA19-9 (U/mL, IQR)		5.4 (13.0–32.9)

*IQR, interquartile range*.

### Comparisons of Gene Mutation Characteristics Between FFPE and Fresh Frozen Tissue

The median and interquartile of total coverage across all genes were 3739 (2148–5866) reads for fresh frozen tissues, which were significantly higher than those of FFPE tissues [2,814 (1,784–3,936) reads] (*P* < 0.001). In our study, 117 patients (99.2%) had one or more variants, with 226 variants in FFPE tissues and 221 in fresh frozen tissues. All variants identified in the FFPE and fresh frozen tissues are showed in Supplemental Table 1.

**Figure 1:**
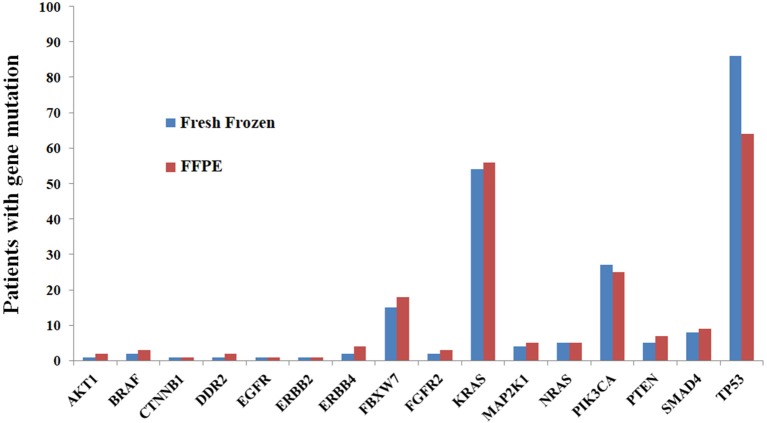
Comparisons of mutation results between FFPE and fresh frozen tissue at the gene level, showing high concordance.

FFPE tissue analysis revealed at least one variant in 112 patients (94.9%), yielding a total of 226 variants. Among 112 patients, 44, 39, 19, 7, 2, and 1 had one, two, three, four, six and seven variants, respectively. The genes mutated most frequently were *TP53* (54.2%), *KRAS* (47.5%), *PIK3CA* (21.2%), and *FBXW7* (15.3%). No mutations were identified in *ALK, FGFR1, FGFR3, MET, NOTCH1, or STK11* (Figure 1).

The analysis of fresh frozen tissue revealed the presence of at least one variant in 105 patients (89.0%), yielding a total of 221 variants. Among the 105 patients, 39, 36, 18, 5, 6, and 1 patients had one, two, three, four, five and six variants, respectively. The list of most frequently mutated genes was similar to that developed for FFPE tissues: *TP53* (72.9%), *KRAS* (45.8%), *PIK3CA* (22.9%), and *FBXW7* (12.7%). No mutations were identified in *ALK, FGFR1, FGFR3, MET, NOTCH1, or STK11* (Figure 1).

A comparison between FFPE and fresh frozen tissue in terms of variant characteristics and their clinical significance was shown in Table 4. The impact of all variants were evaluated using the Variant Impact Predictor Database (VIPdb). No obvious difference in variant impact was found between the two samples (Table 4).

**Table 4:** Comparison of fresh frozen and FFPE tissues in terms of variant characteristics.

	**FFPE tissue**	**Fresh frozen tissue**
Total variants	226	221
**Consequence of variants**		
Missense variant	195	192
Stop gained	19	17
Frameshift variant	5	5
Splice variant	5	4
Inframe deletion	2	3
Inframe insertion	0	0
**Impact of variants**		
Moderate	197	195
High	29	26
**Variant type**		
SNV^*^	216	211
Deletion	8	7
Insertion	2	3
**SIFT analysis**		
Deleterious	159	170
Tolerated	36	22
Unknown	31	29
**PolyPhen-2 analysis**		
Benign	56	51
Probably damaging	114	120
Possibly damaging	25	21
Unknown	31	29
**Clinical significances**		
Likely benign	3	0
Likely pathogenic	8	17
Pathogenic	63	87
Uncertain significance	10	17
Unknown	142	100

* *SNV, single-nucleotide variant (single nucleotide replacement); single nucleotide deletions and insertions were classified separately*.

### Comparison of Gene Mutations Between FFPE and Fresh Frozen Tissue at the Variant Level

A total of 129 variants were identified in this study. Among these variants, 96 were present in both FFPE and fresh frozen tissue; 27 variants were present in FFPE tissues alone; 6 variants were present in fresh frozen tissue alone. Of the 27 variants that existed in FFPE only, 59.3% (16/27) were C>T/G>A or A>G/T>C transitions, 14.8%(4/27) were G>T/T>G transversions, 7.4%(2/27) were A>C transversions, 7.4% (2/27) were A>T/T>A transversions, 3.7%(1/27) was C>G transversion and 7.4% (2/27) were deletion. Among the 6 variants that existed in fresh frozen tissue only, 83.3% (5/6) were C>T/G>A or A>G/T>C transitions, and 16.7% (1/6) was A>C transversion. Comparison of the gene mutations at the variant level revealed that concordance rates were 100.0% for 38.0% (49/129) variants, and concordance rates were higher than 94.0% for all variants (Supplemental Table 2). Kappa coefficients were higher than 0.500 in 64.3% (83/129) of variants (Supplemental Table 2).

### Comparison of Gene Mutations Between FFPE and Fresh Frozen Tissue at the Gene Level

The concordance rates of gene mutations ranged from 73.8 to 100.0% for all genes (Table 5, Figure 1). Kappa coefficients were >0.500 in 81.3% (13/16) of genes (Table 5).

**Table 5:** Concordance of mutation results between fresh frozen and FFPE tissues, at the gene level.

**Mutation Genes**	a^*^	b^*^	c^*^	d^*^	**Concordance^#^**	**Kappa**	***P***
AKT1	1	0	1	116	99.2%	0.663	<0.001
BRAF	1	0	2	116	99.2%	0.796	<0.001
CTNNB1	0	0	1	118	100.0%	1.000	<0.001
DDR2	1	0	1	116	99.2%	0.663	<0.001
EGFR	1	1	0	114	98.3%	−0.009	0.925
ERBB2	0	0	1	105	100.0%	1.000	<0.001
ERBB4	2	1	2	113	97.5%	0.559	<0.001
FBXW7	5	3	13	64	90.6%	0.706	<0.001
FGFR2	1	0	2	113	99.1%	0.796	<0.001
KRAS	16	8	46	92	85.2%	0.679	<0.001
MAP2K1	1	0	4	99	99.0%	0.884	<0.001
NRAS	3	3	2	106	94.7%	0.372	<0.001
PIK3CA	10	12	18	90	83.1%	0.512	<0.001
PTEN	5	2	6	57	90.0%	0.575	<0.001
SMAD4	5	2	6	111	94.4%	0.602	<0.001
TP53	10	35	54	73	73.8%	0.481	<0.001

* a, Mutation identified in fresh frozen tissue, but not in FFPE tissue; b, Mutation identified in FFPE tissue, but not in fresh frozen tissue; c, Mutation identified in FFPE and fresh frozen tissue; d, Mutation was not identified in FFPE or fresh frozen tissue;

# *Concordance = (c+d)/(a+b+c+d)*.

## Discussion

In the present study, we found that 117 patients (99.2%) had one or more variants, with 226 variants in FFPE tissues and 221 in fresh frozen tissues. The concordance rates of gene mutation results in FFPE vs. fresh frozen tissue were higher than 94% for all variants, with Kappa coefficient >0.500 in 64.3% (83/129) variants. At the gene level, concordance ranged from 73.8 to 100.0%, with Kappa coefficient >0.500 in 81.3% (13/16) of genes. Our results indicated that the mutation results for FFPE and fresh frozen tissue were highly concordant at the variant and gene levels, but there were still some important differences in mutation results between the two tissue types.

The total coverage of fresh frozen tissues was significantly higher than that of FFPE tissues. We hypothesized that this could be due to the smaller DNA fragment size in FFPE tissues as some were below the detection limit. In our study, 226 variants were identified in FFPE tissues and 221 variants were identified in fresh frozen tissues. The more variants detected in FFPE samples might be caused by DNA damage during the formalin fixation process (e.g., fragmentation, degradation, crosslinking). Of the 27 variants identified only in FFPE tissues, 16 (59.3%) were C>T/G>A or A>G/T>C transitions. These 16 transitions may be artifacts secondary to postmortem deamination of cytosine or adenine to uracil or hypoxanthine residues (20). We took special care to decrease the rate of false-negative results by including only sections with >40% tumor cells, because adjacent normal cells usually have no mutations (20). In a study by Gallegos et al., the authors compared FFPE samples with fresh frozen tissue samples from 47 lung cancer patients in terms of EGFR and KRAS mutations. The authors showed that the success rate of PCR amplification was only 50% in FFPE tissues, with a false-positive rate up to 50% (19). The high false-positive rate may be related to tissue type and fixation method (18). The fixation and archiving processes required for FFPE often lead to the degradation and fragmentation of DNA. In our study, all PCR primers were designed to make sure that the PCR products were <200 bp, which may have contributed to the high concordance observed.

A few small studies previously compared paired FFPE and fresh frozen tissue from cancer patients in terms of microRNA expression, gene expression, and DNA methylation (24–27). De et al. compared the results of whole-exome sequencing in ten matched FFPE and fresh frozen tissue samples from patients with melanoma (28), and found that the average concordance rate was 43.2% over a total of 1,299 variants for the chosen 27 genes (28). The low concordance rate may be related to the length of PCR product. Spencer et al. compared the variants of 27 cancer-related genes between 16 pairs of fresh frozen and FFPE tissues from patients with lung carcinoma (29), and found that the concordance rate was up to 96.8% in the single-nucleotide variants. Solassol et al. compared the mutation status of *KRAS* in 33 patients with metastatic CRC, using paired fresh frozen and FFPE tumor tissues. The findings obtained revealed a concordance rate of only 81.9% (20). Betge et al. studied gene mutations in paired FFPE and fresh frozen tissue samples from hepatic metastases from 10 patients with CRC. The results revealed a high concordance between samples, with 21 identical variants and 2 different variants (22). All of the above-mentioned studies used NGS to detect mutation, but the testing method were different. The various concordance rates may be related to the various factors such as primer, length of PCR product, tumor type, and testing methods. These results proved that remarkable differences existed between results of FFPE tissues and fresh frozen tissues, which were consistent with our finding. To the best of our knowledge, this is the first study of its kind to systematically compare the rate of mutation in paired fresh frozen and FFPE CRC tissue samples, at the gene and variant levels. Furthermore, our study had a relatively large sample size (118 patients), compared with previous relevant studies.

Although fresh frozen tissue is the gold standard for molecular analyses, its use in clinical practice is impractical because of its high cost and technical difficulty (20). Based on the results of this study, we suggest that archived tissues from pathology departments may be used for mutation detection with the 22-gene panel. The feasibility of using FFPE tissues for mutation detection will facilitate future studies of CRC. However, because the accuracy of mutation detection in FFPE tissues is influenced by multiple factors, it is important to standardize the procedure in order to minimize variability. Standardized protocols should be elaborated for sample preparation, storage room requirements, library preparation, evaluating the quality of extracted DNA, and the exclusion of poor-quality samples (22).

This study had some limitations. First, it was a retrospective study of stored FFPE tissues. In clinical practice, the fixation and embedding of specimens (which includes tissue thickness, fixative volume, and fixation time of 24–72 h) was not strictly controlled. Variation in the above factors may have affected the quality of preserved DNA, leading to inaccurate results (20). Second, the processes of fixation and embedding may result in deamination, leading to artifactual mutations or false-negative results. These factors may have had variable impacts at different tumor sites (20). In this study, we only took 10 consecutive sections of FFPE tumor tissues. We were therefore unable to rule out the possibility of intra-tumor heterogeneity in terms of DNA deamination. Third, although it is the largest study of its kind, larger prospective studies are required to validate our results. The number of variants will increase with sample size, leading to a more accurate evaluation of concordance, which is especially important for rare variants. Fourth, our study included FFPE specimens that had been stored in the pathology department for <2 years. The degradation and fragmentation of DNA in FFPE tissues may increase with time. Therefore, our observations cannot be extrapolated to all FFPE specimens.

## Conclusion

The gene mutation results of a 22-gene panel showed high concordance between paired FFPE and fresh frozen tissue samples, at both the variant and gene levels. This indicates that FFPE tissues stored for <2 years may be used as an alternative to fresh frozen tissue for detecting gene mutations in patients with CRC. However, any alteration in the preparation or detection process may affect the accuracy of results. Factors that may be affected include fixation time, duration of storage of FFPE specimens, DNA sample quality, and tools used to analyze the variants. This should be taken into consideration when interpreting the findings presented above. Furthermore, the mutation results still showed some differences between tissues. Therefore, fresh frozen tissue should not routinely be replaced with FFPE for mutation analysis with a multi-gene panel; instead, FFPE is a reasonable alternative for fresh frozen tissue when the latter is unavailable. In addition, if the clinical response of *EGFR*-targeted therapy were not consistent with the mutation results based on FFPE tissue, gene mutation test might be performed again with fresh frozen tissue.

## Data Availability Statement

The raw data supporting the conclusions of this article can be found in the supplementary files, and are available on request to the corresponding author.

## Ethics Statement

The studies involving human participants were reviewed and approved by the ethical committee at Changhai Hospital. The patients/participants provided their written informed consent to participate in this study.

## Author Contributions

LL, CB, and WZ: conceptualization. XG, JL, and HG: data curation and writing—original draft. XG and JL: formal analysis. XG: funding acquisition. HG, GY, and PL: investigation. LH: methodology. CB: resources. LL and WZ: supervision. GY: validation. GY, PL, LH, LL, CB, and WZ: writing—review and editing.

### Conflict of Interest

The authors declare that the research was conducted in the absence of any commercial or financial relationships that could be construed as a potential conflict of interest.
