# Supplementary material for: Comparison of Fresh Frozen Tissue With Formalin-Fixed Paraffin-Embedded Tissue for Mutation Analysis Using a Multi-Gene Panel in Patients With Colorectal Cancer
Source: Front Oncol. 2020 Mar 13;10:310. doi: 10.3389/fonc.2020.00310 (PMC7083147; doi:10.3389/fonc.2020.00310)
Supplement: Supplementary file 2 [file Table_2.docx]

**Supplemental table 2.** Concordance of mutation results between fresh frozen and FFPE tissues at variant level

| No. | Variants | a* | b* | c* | d* | Concordance# | Kappa | P |
| --- | --- | --- | --- | --- | --- | --- | --- | --- |
| 1 | AKT1+c.49G>A | 1 | 0 | 1 | 116 | 99.2% | 0.663 | <0.001 |
| 2 | BRAF+c.1799T>A | 1 | 0 | 2 | 115 | 99.2% | 0.796 | <0.001 |
| 3 | CTNNB1+c.134C>T | 0 | 0 | 1 | 117 | 100.0% | 1.000 | 0.000 |
| 4 | DDR2+c.1891C>T | 0 | 0 | 1 | 117 | 100.0% | 1.000 | 0.000 |
| 5 | DDR2+c.1970T>C | 1 | 0 | 0 | 117 | 99.2% | - | - |
| 6 | EGFR+c.2155G>C | 1 | 1 | 0 | 116 | 98.3% | -0.009 | 0.926 |
| 7 | ERBB2+c.2264T>C | 0 | 0 | 1 | 117 | 100.0% | 1.000 | 0.000 |
| 8 | ERBB4+c.1834C>T | 0 | 1 | 0 | 117 | 99.2% | - | - |
| 9 | ERBB4+c.340C>T | 1 | 0 | 1 | 116 | 99.2% | 0.663 | <0.001 |
| 10 | ERBB4+c.411G>T | 1 | 0 | 1 | 116 | 99.2% | 0.663 | <0.001 |
| 11 | FBXW7+c.1390G>A | 0 | 0 | 1 | 117 | 100.0% | 1.000 | 0.000 |
| 12 | FBXW7+c.1393C>T | 0 | 0 | 1 | 117 | 100.0% | 1.000 | 0.000 |
| 13 | FBXW7+c.1394G>A | 1 | 0 | 3 | 114 | 99.2% | 0.853 | <0.001 |
| 14 | FBXW7+c.1394G>T | 0 | 0 | 1 | 117 | 100.0% | 1.000 | 0.000 |
| 15 | FBXW7+c.1436G>A | 1 | 1 | 3 | 113 | 98.3% | 0.741 | <0.001 |
| 16 | FBXW7+c.1513C>T | 1 | 0 | 2 | 115 | 99.2% | 0.796 | <0.001 |
| 17 | FBXW7+c.1738C>T | 0 | 1 | 0 | 117 | 99.2% | - | - |
| 18 | FBXW7+c.1745C>T | 1 | 0 | 0 | 117 | 99.2% | - | - |
| 19 | FBXW7+c.1774A>G | 0 | 0 | 1 | 117 | 100.0% | 1.000 | 0.000 |
| 20 | FBXW7+c.832C>T | 1 | 1 | 1 | 115 | 98.3% | 0.491 | <0.001 |
| 21 | FGFR2+c.1611G>A | 0 | 0 | 1 | 117 | 100.0% | 1.000 | 0.000 |
| 22 | FGFR2+c.811G>A | 1 | 0 | 1 | 116 | 99.2% | 0.663 | <0.001 |
| 23 | KRAS+c.175G>A | 2 | 0 | 0 | 116 | 98.3% | 0.000 | 1.000 |
| 24 | KRAS+c.179G>A | 1 | 0 | 1 | 116 | 99.2% | 0.663 | <0.001 |
| 25 | KRAS+c.183A>C | 0 | 1 | 0 | 117 | 99.2% | - | - |
| 26 | KRAS+c.34G>A | 3 | 0 | 2 | 113 | 97.5% | 0.561 | <0.001 |
| 27 | KRAS+c.34G>C | 0 | 0 | 2 | 116 | 100.0% | 1.000 | 0.000 |
| 28 | KRAS+c.34G>T | 1 | 2 | 3 | 112 | 97.5% | 0.654 | <0.001 |
| 29 | KRAS+c.350A>G | 0 | 0 | 1 | 117 | 100.0% | 1.000 | 0.000 |
| 30 | KRAS+c.35G>A | 3 | 1 | 21 | 93 | 96.6% | 0.892 | <0.001 |
| 31 | KRAS+c.35G>C | 1 | 1 | 1 | 115 | 98.3% | 0.491 | <0.001 |
| 32 | KRAS+c.35G>T | 1 | 2 | 5 | 110 | 97.5% | 0.756 | <0.001 |
| 33 | KRAS+c.38G>A | 4 | 1 | 7 | 106 | 95.8% | 0.714 | <0.001 |
| 34 | KRAS+c.436G>A | 0 | 0 | 3 | 115 | 100.0% | 1.000 | 0.000 |
| 35 | MAP2K1+c.139C>T | 1 | 0 | 1 | 116 | 99.2% | 0.663 | <0.001 |
| 36 | MAP2K1+c.145C>T | 0 | 0 | 2 | 116 | 100.0% | 1.000 | 0.000 |
| 37 | MAP2K1+c.171G>T | 0 | 0 | 1 | 117 | 100.0% | 1.000 | 0.000 |
| 38 | NRAS+c.182A>G | 0 | 1 | 0 | 117 | 99.2% | - | - |
| 39 | NRAS+c.182A>T | 1 | 0 | 0 | 117 | 99.2% | - | - |
| 40 | NRAS+c.35G>A | 2 | 1 | 1 | 114 | 97.5% | 0.388 | <0.001 |
| 41 | NRAS+c.38G>T | 0 | 0 | 1 | 117 | 100.0% | 1.000 | 0.000 |
| 42 | NRAS+c.52G>A | 0 | 1 | 0 | 117 | 99.2% | - | - |
| 43 | PIK3CA+c.1220G>T | 1 | 0 | 1 | 116 | 99.2% | 0.663 | <0.001 |
| 44 | PIK3CA+c.1258T>C | 1 | 2 | 0 | 115 | 97.5% | -0.011 | 0.895 |
| 45 | PIK3CA+c.1624G>A | 1 | 0 | 2 | 115 | 99.2% | 0.796 | <0.001 |
| 46 | PIK3CA+c.1633G>A | 2 | 2 | 6 | 108 | 96.6% | 0.732 | <0.001 |
| 47 | PIK3CA+c.1636C>G | 0 | 0 | 1 | 117 | 100.0% | 1.000 | 0.000 |
| 48 | PIK3CA+c.1637A>C | 0 | 0 | 1 | 117 | 100.0% | 1.000 | 0.000 |
| 49 | PIK3CA+c.1647T>A | 0 | 0 | 1 | 117 | 100.0% | 1.000 | 0.000 |
| 50 | PIK3CA+c.263G>A | 1 | 0 | 2 | 115 | 99.2% | 0.796 | <0.001 |
| 51 | PIK3CA+c.2727C>A | 0 | 0 | 1 | 117 | 100.0% | 1.000 | 0.000 |
| 52 | PIK3CA+c.278G>T | 0 | 1 | 0 | 117 | 99.2% | - | - |
| 53 | PIK3CA+c.3061T>C | 0 | 1 | 0 | 117 | 99.2% | - | - |
| 54 | PIK3CA+c.3139C>T | 0 | 1 | 0 | 117 | 99.2% | - | - |
| 55 | PIK3CA+c.3140A>G | 1 | 3 | 0 | 114 | 96.6% | -0.013 | 0.871 |
| 56 | PIK3CA+c.3140A>T | 0 | 1 | 0 | 117 | 99.2% | - | - |
| 57 | PIK3CA+c.317G>A | 1 | 0 | 1 | 116 | 99.2% | 0.663 | <0.001 |
| 58 | PIK3CA+c.3203dupA | 1 | 0 | 1 | 116 | 99.2% | 0.663 | <0.001 |
| 59 | PIK3CA+c.331A>G | 0 | 0 | 1 | 117 | 100.0% | 1.000 | 0.000 |
| 60 | PIK3CA+c.959G>A | 1 | 1 | 0 | 116 | 98.3% | -0.009 | 0.926 |
| 61 | PTEN+c.19G>T | 1 | 0 | 1 | 116 | 99.2% | 0.663 | <0.001 |
| 62 | PTEN+c.209+1_209+2del | 0 | 0 | 1 | 117 | 100.0% | 1.000 | 0.000 |
| 63 | PTEN+c.32G>T | 1 | 0 | 1 | 116 | 99.2% | 0.663 | <0.001 |
| 64 | PTEN+c.382A>G | 1 | 0 | 1 | 116 | 99.2% | 0.663 | <0.001 |
| 65 | PTEN+c.517C>T | 0 | 0 | 1 | 117 | 100.0% | 1.000 | 0.000 |
| 66 | PTEN+c.518G>A | 0 | 1 | 0 | 117 | 99.2% | - | - |
| 67 | PTEN+c.708dupC | 1 | 1 | 0 | 116 | 98.3% | -0.009 | 0.926 |
| 68 | PTEN+c.734A>C | 1 | 0 | 0 | 117 | 99.2% | - | - |
| 69 | PTEN+c.800delA | 0 | 0 | 1 | 117 | 100.0% | 1.000 | 0.000 |
| 70 | SMAD4+c.1082G>A | 2 | 1 | 3 | 112 | 97.5% | 0.654 | <0.001 |
| 71 | SMAD4+c.1156G>C | 0 | 0 | 1 | 117 | 100.0% | 1.000 | 0.000 |
| 72 | SMAD4+c.1511_1513delGT | 1 | 0 | 1 | 116 | 99.2% | 0.663 | <0.001 |
| 73 | SMAD4+c.319A>C | 0 | 1 | 0 | 117 | 99.2% | - | - |
| 74 | SMAD4+c.353C>T | 1 | 0 | 0 | 117 | 99.2% | - | - |
| 75 | SMAD4+c.386A>G | 1 | 0 | 1 | 116 | 99.2% | 0.663 | <0.001 |
| 76 | TP53+c.1009C>T | 0 | 1 | 0 | 117 | 99.2% | - | - |
| 77 | TP53+c.1015G>T | 0 | 0 | 1 | 117 | 100.0% | 1.000 | 0.000 |
| 78 | TP53+c.1024C>T | 0 | 1 | 2 | 115 | 99.2% | 0.796 | <0.001 |
| 79 | TP53+c.376-1G>T | 0 | 0 | 1 | 117 | 100.0% | 1.000 | 0.000 |
| 80 | TP53+c.376-4_379delAC | 1 | 1 | 0 | 116 | 98.3% | -0.009 | 0.926 |
| 81 | TP53+c.403T>C | 0 | 0 | 1 | 117 | 100.0% | 1.000 | 0.000 |
| 82 | TP53+c.404G>A | 0 | 0 | 1 | 117 | 100.0% | 1.000 | 0.000 |
| 83 | TP53+c.451C>T | 0 | 1 | 0 | 117 | 99.2% | - | - |
| 84 | TP53+c.455C>T | 0 | 0 | 1 | 117 | 100.0% | 1.000 | 0.000 |
| 85 | TP53+c.470T>A | 0 | 1 | 0 | 117 | 99.2% | - | - |
| 86 | TP53+c.473G>A | 0 | 1 | 0 | 117 | 99.2% | - | - |
| 87 | TP53+c.476C>T | 0 | 0 | 1 | 117 | 100.0% | 1.000 | 0.000 |
| 88 | TP53+c.488A>G | 0 | 0 | 1 | 117 | 100.0% | 1.000 | 0.000 |
| 89 | TP53+c.493C>T | 0 | 2 | 0 | 116 | 98.3% | 0.000 | 1.000 |
| 90 | TP53+c.497C>G | 0 | 1 | 0 | 117 | 99.2% | - | - |
| 91 | TP53+c.514G>T | 0 | 0 | 1 | 117 | 100.0% | 1.000 | 0.000 |
| 92 | TP53+c.517G>A | 0 | 1 | 0 | 117 | 99.2% | - | - |
| 93 | TP53+c.518T>G | 0 | 0 | 1 | 117 | 100.0% | 1.000 | 0.000 |
| 94 | TP53+c.519_551delGAGG | 0 | 0 | 1 | 117 | 100.0% | 1.000 | 0.000 |
| 95 | TP53+c.524G>A | 0 | 7 | 3 | 108 | 94.1% | 0.440 | <0.001 |
| 96 | TP53+c.526T>C | 0 | 1 | 0 | 117 | 99.2% | - | - |
| 97 | TP53+c.527G>T | 0 | 0 | 1 | 117 | 100.0% | 1.000 | 0.000 |
| 98 | TP53+c.535C>T | 1 | 1 | 0 | 116 | 98.3% | -0.009 | 0.926 |
| 99 | TP53+c.537T>G | 0 | 1 | 0 | 117 | 99.2% | - | - |
| 100 | TP53+c.541C>T | 0 | 1 | 0 | 117 | 99.2% | - | - |
| 101 | TP53+c.559+1delG | 0 | 1 | 0 | 117 | 99.2% | - | - |
| 102 | TP53+c.578A>G | 0 | 0 | 1 | 117 | 100.0% | 1.000 | 0.000 |
| 103 | TP53+c.581T>G | 0 | 0 | 2 | 116 | 100.0% | 1.000 | 0.000 |
| 104 | TP53+c.584T>C | 1 | 2 | 2 | 113 | 97.5% | 0.559 | <0.001 |
| 105 | TP53+c.586C>T | 0 | 0 | 2 | 116 | 100.0% | 1.000 | 0.000 |
| 106 | TP53+c.604C>T | 0 | 1 | 0 | 117 | 99.2% | - | - |
| 107 | TP53+c.614A>G | 0 | 0 | 1 | 117 | 100.0% | 1.000 | 0.000 |
| 108 | TP53+c.637C>T | 0 | 0 | 4 | 114 | 100.0% | 1.000 | 0.000 |
| 109 | TP53+c.646G>A | 0 | 0 | 1 | 117 | 100.0% | 1.000 | 0.000 |
| 110 | TP53+c.657delC | 0 | 0 | 1 | 117 | 100.0% | 1.000 | 0.000 |
| 111 | TP53+c.659A>G | 1 | 0 | 2 | 115 | 99.2% | 0.796 | <0.001 |
| 112 | TP53+c.673-1G>T | 0 | 0 | 1 | 117 | 100.0% | 1.000 | 0.000 |
| 113 | TP53+c.701A>G | 0 | 0 | 3 | 115 | 100.0% | 1.000 | 0.000 |
| 114 | TP53+c.713G>A | 0 | 0 | 1 | 117 | 100.0% | 1.000 | 0.000 |
| 115 | TP53+c.733G>A | 0 | 1 | 2 | 115 | 99.2% | 0.796 | <0.001 |
| 116 | TP53+c.742C>T | 0 | 1 | 1 | 116 | 99.2% | 0.663 | <0.001 |
| 117 | TP53+c.743G>A | 0 | 0 | 5 | 113 | 100.0% | 1.000 | 0.000 |
| 118 | TP53+c.743G>T | 0 | 1 | 0 | 117 | 99.2% | - | - |
| 119 | TP53+c.746G>T | 1 | 0 | 1 | 116 | 99.2% | 0.663 | <0.001 |
| 120 | TP53+c.772G>A | 0 | 0 | 1 | 117 | 100.0% | 1.000 | 0.000 |
| 121 | TP53+c.772G>T | 0 | 1 | 0 | 117 | 99.2% | - | - |
| 122 | TP53+c.808T>G | 0 | 0 | 1 | 117 | 100.0% | 1.000 | 0.000 |
| 123 | TP53+c.814G>T | 0 | 0 | 1 | 117 | 100.0% | 1.000 | 0.000 |
| 124 | TP53+c.815T>G | 0 | 0 | 1 | 117 | 100.0% | 1.000 | 0.000 |
| 125 | TP53+c.817C>T | 3 | 1 | 0 | 114 | 96.6% | -0.013 | 0.871 |
| 126 | TP53+c.818G>A | 1 | 3 | 1 | 113 | 96.6% | 0.318 | <0.001 |
| 127 | TP53+c.825_826delTG | 0 | 1 | 0 | 117 | 99.2% | - | - |
| 128 | TP53+c.833C>T | 0 | 1 | 0 | 117 | 99.2% | - | - |
| 129 | TP53+c.844C>T | 1 | 0 | 3 | 114 | 99.2% | 0.853 | <0.001 |

*a: mutation was identified in fresh frozen tissue, but not in FFPE tissue; b: mutation was identified in FFPE tissue, but not in fresh frozen tissue; c: mutation was identified in both FFPE and fresh frozen tissue; d: mutation was not identified in FFPE or fresh frozen tissue; #Concordance =(c+d)/(a+b+c+d).
